# The Economic Value of Long-Lasting Insecticidal Nets and Indoor Residual Spraying Implementation in Mozambique

**DOI:** 10.4269/ajtmh.16-0744

**Published:** 2017-06-07

**Authors:** Bruce Y. Lee, Sarah M. Bartsch, Nathan T. B. Stone, Shufang Zhang, Shawn T. Brown, Chandrani Chatterjee, Jay V. DePasse, Eli Zenkov, Olivier J. T. Briët, Chandana Mendis, Kirsi Viisainen, Baltazar Candrinho, James Colborn

**Affiliations:** 1 Johns Hopkins Bloomberg School of Public Health, Baltimore, Maryland; 2 Pittsburgh Supercomputing Center, Carnegie Mellon University, Pittsburgh, Pennsylvania; 3 The Global Fund to Fight AIDS, Tuberculosis, and Malaria, Geneva, Switzerland; 4 Swiss Tropical and Public Health Institute, Basel, Switzerland; 5 University of Basel, Basel, Switzerland; 6 World Vision International, Maputo, Mozambique; 7 National Malaria Control Program, Mozambique Ministry of Health, Maputo, Mozambique; 8 President's Malaria Initiative, Centers for Disease Control and Prevention, Washington, District of Columbia

## Abstract

Malaria-endemic countries have to decide how much of their limited resources for vector control to allocate toward implementing long-lasting insecticidal nets (LLINs) versus indoor residual spraying (IRS). To help the Mozambique Ministry of Health use an evidence-based approach to determine funding allocation toward various malaria control strategies, the Global Fund convened the Mozambique Modeling Working Group which then used JANUS, a software platform that includes integrated computational economic, operational, and clinical outcome models that can link with different transmission models (in this case, OpenMalaria) to determine the economic value of vector control strategies. Any increase in LLINs (from 80% baseline coverage) or IRS (from 80% baseline coverage) would be cost-effective (incremental cost-effectiveness ratios ≤ $114/disability-adjusted life year averted). However, LLIN coverage increases tend to be more cost-effective than similar IRS coverage increases, except where both pyrethroid resistance is high and LLIN usage is low. In high-transmission northern regions, increasing LLIN coverage would be more cost-effective than increasing IRS coverage. In medium-transmission central regions, changing from LLINs to IRS would be more costly and less effective. In low-transmission southern regions, LLINs were more costly and less effective than IRS, due to low LLIN usage. In regions where LLINs are more cost-effective than IRS, it is worth considering prioritizing LLIN coverage and use. However, IRS may have an important role in insecticide resistance management and epidemic control. Malaria intervention campaigns are not a one-size-fits-all solution, and tailored approaches are necessary to account for the heterogeneity of malaria epidemiology.

## Introduction

In 2015, malaria resulted in an estimated 214 million cases and 438,000 deaths worldwide; 89% of these cases and 91% of these deaths occurred in sub-Saharan Africa.^1^ To address this, the World Health Organization (WHO) has recommended that all persons at risk for malaria should be covered by at least one vector control intervention, either the use of insecticide-treated nets (or long-lasting insecticidal nets [LLINs]) or indoor residual spraying (IRS). Due to the cost of these interventions, most countries in which malaria is endemic have to decide how many resources to allocate toward vector control, and within their budget for vector control, whether to prioritize LLINs or IRS. For example, in 2014, the country of Mozambique was in the midst of developing an application to the Global Fund to support its malaria control programs in which decisions needed to be made on what kinds of vector control would be implemented and where. According to the last national cross-sectional survey in Mozambique (performed in 2011), malaria accounts for 29% of all deaths (42% among children < 5 years of age),^2^ for 40% of all outpatient visits and 60% of pediatric ward admissions.^3^ However, current epidemiologic data show that malaria is on the rise with reported cases increasing by 40% from 2013 to 2014, outpatient visits increasing by 5%, and deaths increasing by 10%.^4^

Currently, there is a relative dearth of evidence-based approaches to determine how funds should be divided between supporting LLINs and IRS. To facilitate decision-making by the Mozambique Ministry of Health, the Global Fund convened the Mozambique Modeling Working Group (MMWG) that used JANUS, a computational economic, operational, and clinical outcomes modeling platform connected with OpenMalaria,^5^ a malaria transmission model to determine the value of changing LLIN and IRS coverage. The goal of the MMWG was to investigate the cost-effectiveness of differing allocation options of LLINs versus IRS, taking into account the heterogeneous transmission throughout the country, to inform the country's Global Fund application. This article describes the findings of the MMWG.

## Methods

### Mozambique Modeling Working Group.

Convened by the Global Fund to Fight AIDS, Tuberculosis and Malaria in 2014, the MMWG consists of Johns Hopkins University, the Pittsburgh Supercomputing Center at Carnegie Mellon University, Swiss Tropical and Public Health Institute, President's Malaria Initiative from the Centers for Disease Control and Prevention, World Vision International in Mozambique, National Malaria Control Program, Ministry of Health in Mozambique, and the London School of Economics.

### JANUS Malaria Mozambique Model.

**Figure 1.**
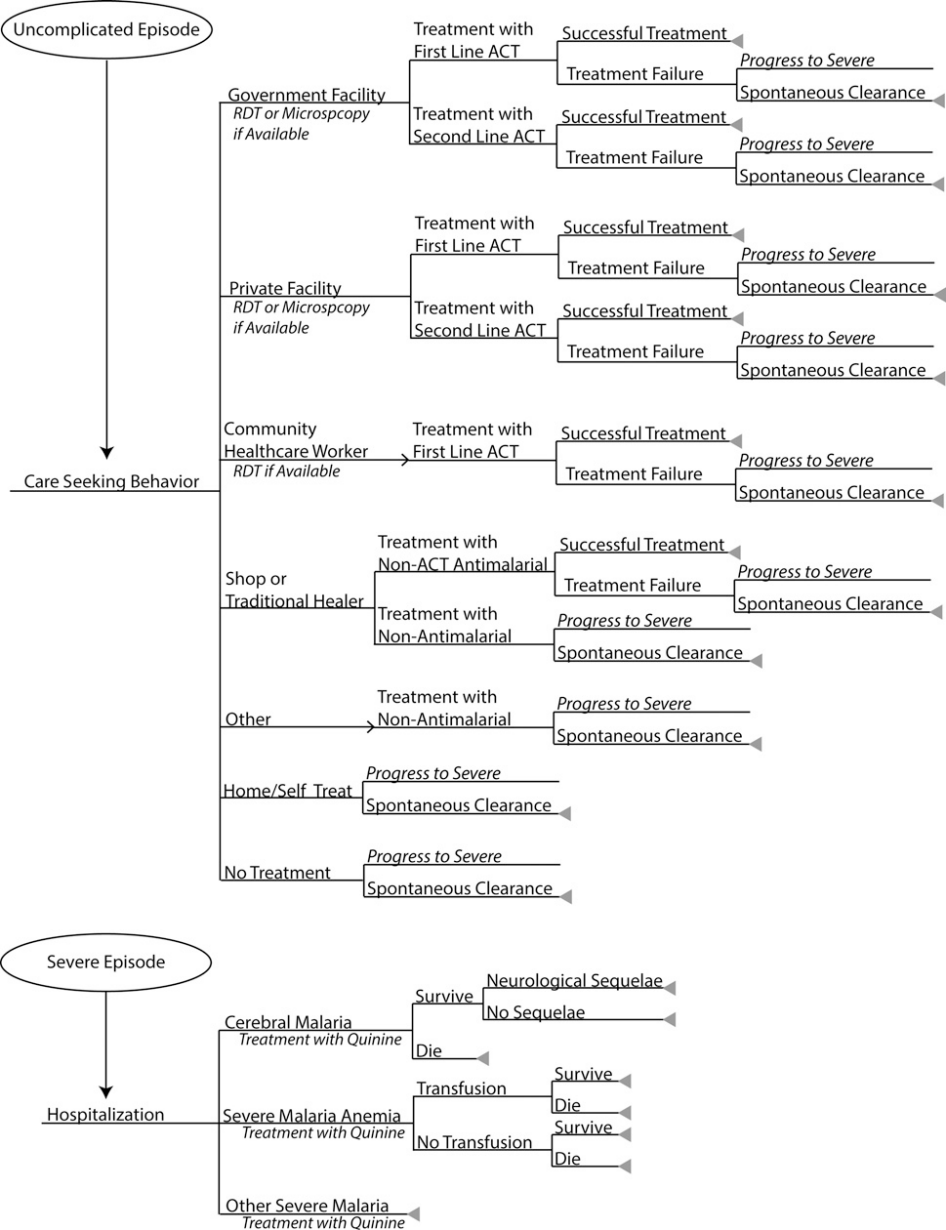
JANUS Malaria clinical outcomes model structure.

JANUS Malaria includes integrated malaria clinical, economic, and operational models, and can connect to malaria transmission models to compare the impact of implementing different portfolios of interventions in a chosen location. Developed in Python/Django, JANUS allows users to select a geographic location, import data on that location, choose what interventions will comprise a portfolio, select the native characteristics (e.g., efficacy, cost, duration of action) of each intervention, and choose how, when, and where each intervention will be implemented (e.g., geospatial coverage, supply and administration costs, and target population over time); it also allows users to run models deterministically or stochastically. Our team has used JANUS in Kenya, Zambia, and Vietnam to evaluate the cost-effectiveness of vector control strategies. The MMWG used JANUS Malaria, connected to the OpenMalaria malaria transmission model, to determine the impact on malaria transmission and generate the number of new malaria cases over time. Supplemental Appendix provides details on JANUS's malaria clinical outcomes model (Figure 1

) and Table 1 provides the model input parameters, values, and data references. Both models were run deterministically since all data inputs were flat values. All data came from country reports, surveys, and databases, international organizations (e.g., WHO and World Bank), and the scientific literature and, when necessary, we supplemented with expert opinion.

### Baseline scenario: current circumstances in Mozambique.

Mozambique's malaria transmissibility, insecticide resistance patterns, and intervention implementation varies by region. Mosquito resistance to insecticides, particularly pyrethroids, is growing and varies by region and vector species.^6^–^9^ We accounted for these resistance patterns and levels to evaluate the impact on the effectiveness of pyrethroid-based LLINs as well as how transmission intensity varies by region. The entomological inoculation rate (EIR) or the number of infectious bites by mosquitos per person per year is the measure of transmission intensity. Following the Malaria Atlas Project,^10^ we used EIR values of 1, 5, and 20 bites per person to represent typical low, medium, and high values, respectively, and represented the following three large regions of Mozambique corresponding to each of these transmission levels:

1. Northern region: high transmission intensity (EIR 20); *Anopheles gambiae* and *Anopheles arabiensis* exhibit high insecticide resistance; 65% LLIN usage conditional on access^11^,^12^
2. Central region: medium transmission intensity (EIR 5); *Anopheles funestus* exhibits medium insecticide resistance; 60% LLIN usage conditional on access^11^,^12^
3. Southern region: low transmission intensity (EIR 1); *A. funestus* exhibits high resistance; 35% LLIN usage conditional on access^12^

IRS scenarios assumed Actellic (pirimphos methyl) CS and accounted for transmission intensity by region (described above). We assumed there was no insecticide resistance as Actellic CS exhibits little to no resistance. Additionally, acceptance of IRS was not explicitly considered as the coverage data inherently accounts for this among those in the targeted population (i.e., in-county coverage estimates account for acceptability of IRS among residents targeted for IRS).

Currently, Mozambique deploys vector control through campaigns at district level. Based on in-country reporting, LLIN coverage varied by year (depending on distribution and attrition) but at any one given time, net coverage is 80% or higher, while, as of 2011, the population covered by IRS (among residents targeted for IRS) was 83%. To be generally representative, we modeled a single, shared historic deployment of IRS or LLINs for a region. For each region, we modeled two baselines: 1) 80% coverage with LLINs and 2) 80% coverage of IRS. Coverage was modeled per 1,000 persons (i.e., we modeled only the targeted population). We assumed LLINs and IRS are distributed every 3 and 1 years, respectively, and determined the cost of each per person protected per distribution. Intervention costs were derived from in-country budget planning and documentation. The LLIN budget included the total product, procurement, and distribution costs down to the local level by the number of nets needed in 2014. The IRS budget included insecticide procurement, supply chain management, implementation, and waste management for DDT costs for Mozambique's IRS implementation plan for 2014, 2015, and 2016. More details are provided in Supplemental Appendix.

### Experimental scenarios and sensitivity analysis.

Different scenarios simulated the epidemiologic and economic impact of progressively increasing LLIN and IRS coverage (from 80% up to 100% for each) under various conditions (e.g., different levels of LLIN usage and insecticide resistance, and LLIN and IRS costs ±20%) in the three regions in Mozambique. Each scenario considered only one intervention at a time (i.e., only LLINs or only IRS). For each scenario, the following formula determined the incremental cost-effectiveness ratio (ICER) compared with continuing the baseline scenario:

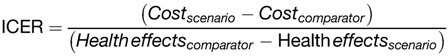
with health effects measured in disability-adjusted life years (DALYs). DALYs equaled the sum of the years lost to disability (YLD) and the years of life lost (YLL) due to premature mortality. The following formulas calculate the YLD and YLL:

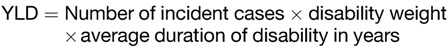


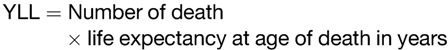


Based on WHO guidelines, a scenario was considered highly cost-effective versus its comparator if the ICERs were less than Mozambique's gross domestic product (GDP) per capita ($608)^13^ and cost-effective if the ICERs were between one and three times the GDP per capita, and not cost-effective if the ICERs were more than three times the GDP per capita ($1,823).

## Results

### Baseline: current situation in Mozambique.

Figure 2

**Figure 2.**
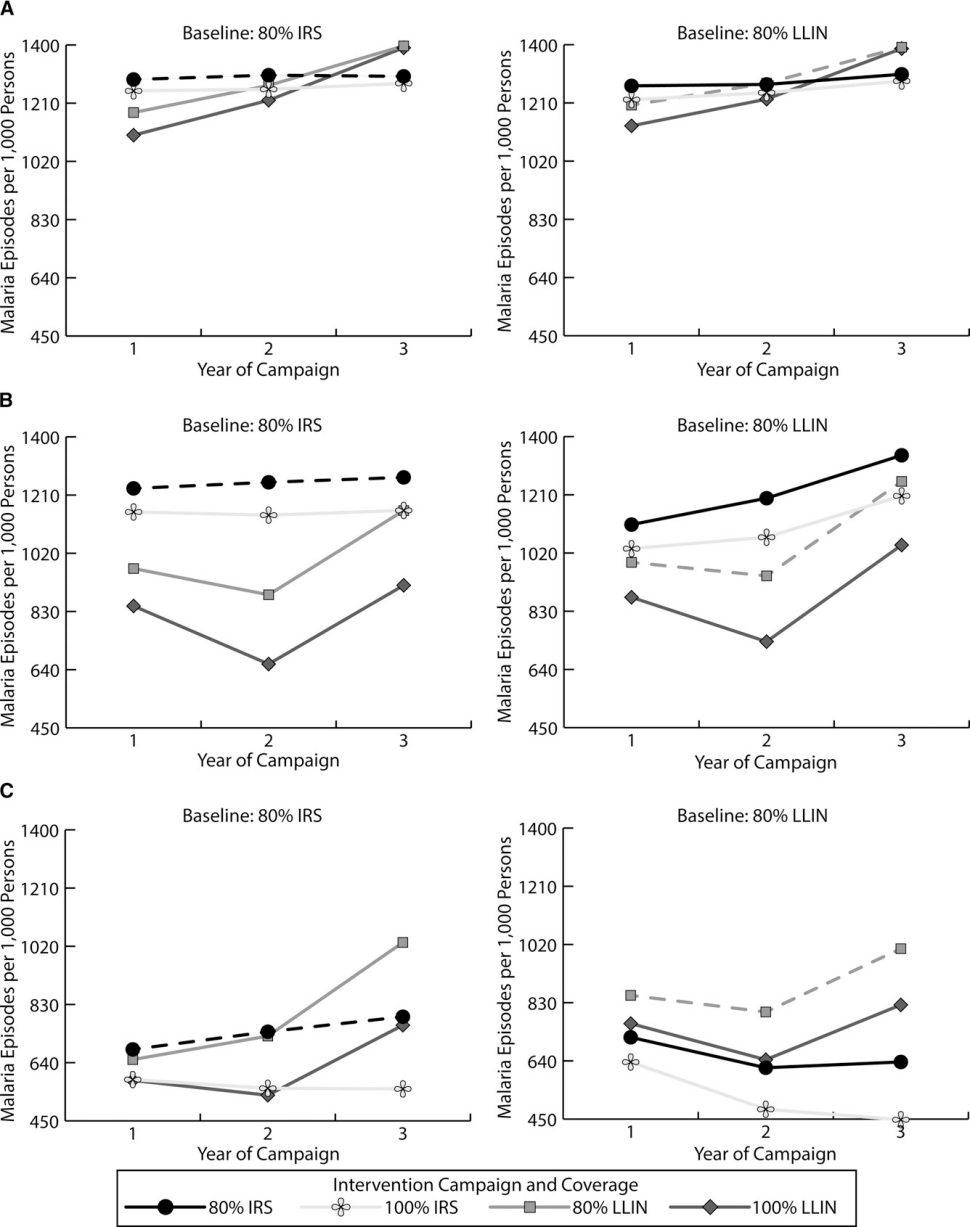
Malaria episodes per 1,000 persons over 3-year campaigns by region in Mozambique ([**A**] northern region; [**B**] central region; and [**C**] southern region) and assumed regional insecticide resistance patterns and long-lasting insecticidal net (LLIN) usage rates. Dotted lines indicate that the current malaria control strategies are maintained.

shows the number of malaria episodes per 1,000 of the population by region. The dotted lines in each panel (representing 80% IRS in the left panels and 80% LLINs in the right panels) plot the number of annual new malaria episodes per 1,000 persons for the next 3 years if the current malaria control strategies in Mozambique were maintained (i.e., there is no change in intervention strategy, initial coverage, or usage). The increase in the number of new malaria episodes in years 2 and 3 with LLIN use reflect their single deployment (in year 1) and their decreasing effectiveness and coverage over time due to “wear and tear.” Figure 3

**Figure 3.**
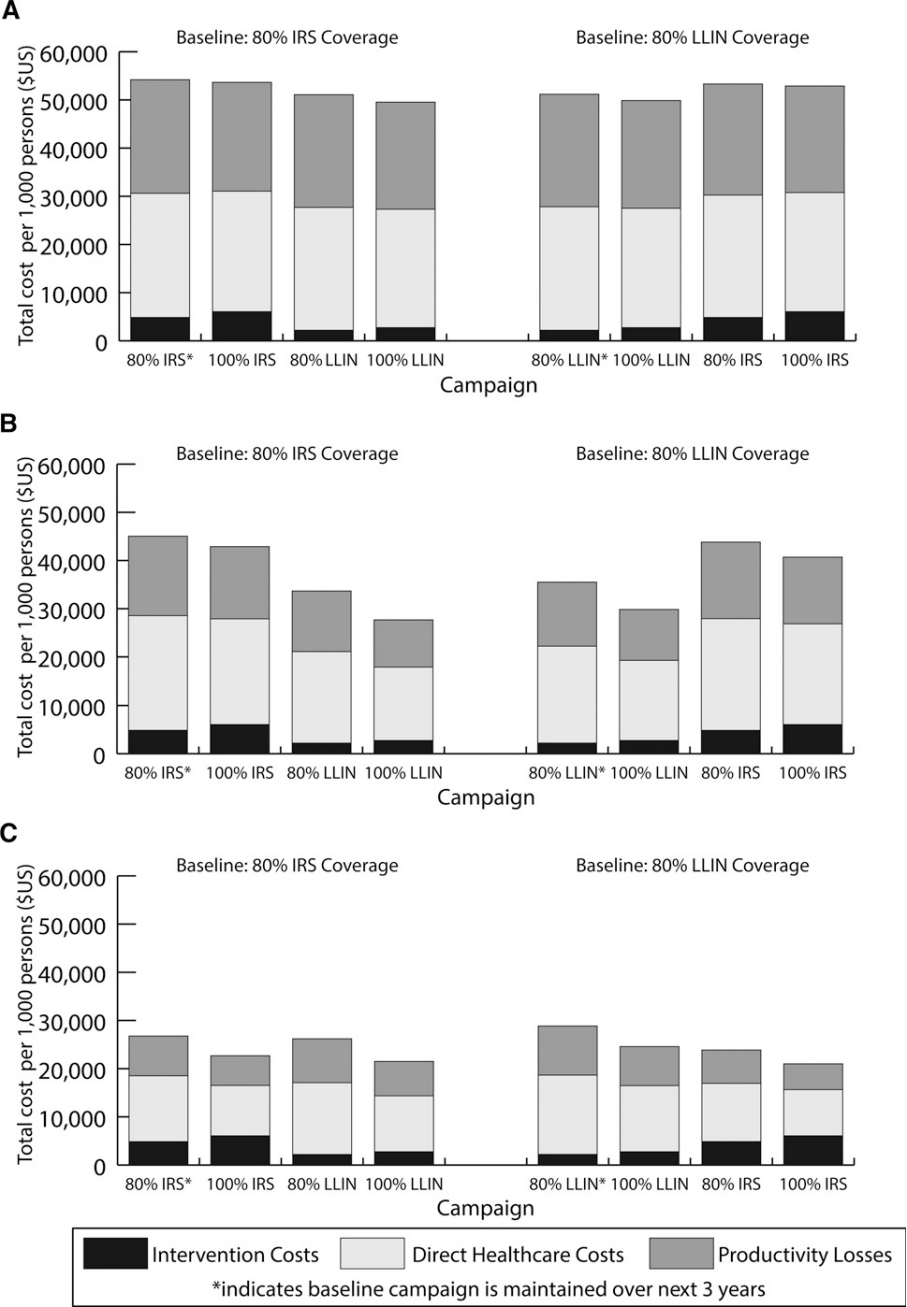
Breakdown of total costs (intervention costs, direct health-care costs, and productivity losses) per 1,000 persons targeted for intervention by region in Mozambique ([**A**] northern region; [**B**] central region; and [**C**] southern region) and assumed regional insecticide resistance patterns and long-lasting insecticidal net (LLIN) usage rates.

shows the total intervention, direct health care (i.e., provider costs), and productivity loss costs over the 3 years if continuing the same control strategy. Intervention costs per 1,000 persons in the targeted population were similar across regions, costing ∼$2,200 for LLINs and ∼$4,850 for IRS, at 80% coverage. In Figure 3, the sum of the intervention costs and direct health-care costs gives the total direct costs, whereas the sum of the entire column yields the total societal costs. For example, in the northern region of Mozambique, over a 3-year period implementing 80% IRS resulted in $25,764 in total direct costs and $49,355 in societal costs per 1,000 persons, whereas implementing 80% LLINs resulted in $25,631 in total direct costs and $48,982 in societal costs per 1,000 persons (Figure 3A).

### Impact of varying LLIN and IRS coverage.

The solid lines in Figure 2 show the number of malaria episodes for changes in intervention campaign (i.e., either increase in coverage or switching strategies) from the baseline intervention assumed before the campaign change (i.e., intervention at year 0) and assume the current usage and resistance patterns in Mozambique. Episodes varied by region and intervention. In northern regions, while LLIN use resulted in fewer episodes over the 3-year period, LLIN campaigns resulted in fewer episodes in year 1 and IRS campaigns resulted in fewer episodes in year 3 (Figure 2A). In central regions, LLIN campaigns tended to consistently result in fewer episodes (Figure 2B). In southern regions, where resistance levels were high and LLIN usage rates low, IRS campaigns tended to result in fewer malaria episodes (Figure 2C).

Table 2 reports the number of malaria episodes, deaths, and DALYs averted for each campaign compared with maintaining Mozambique's current control measures. All evaluated campaigns were effective at reducing Mozambique's malaria burden in the northern region. In the central region, assuming use of LLINs with 60% usage, only increasing coverage to 100% would reduce malaria's burden, and switching to IRS would result in additional episodes, deaths, and DALYs.

Table 3 shows the economic results (ICER and cost per additional episode averted). Negative values imply cost savings. In general, any increase in coverage of the baseline intervention (in both LLIN and IRS scenarios) would be cost-effective (ICERs ≤ $114/DALY averted). However, LLINs tended to be more cost-effective than IRS, except where both pyrethroid resistance was high and LLIN usage was low (e.g., in southern regions of Mozambique). Cost-effectiveness varied by region (Table 3), reflecting the differences in malaria transmission, resistance, and LLIN usage. In the northern regions, LLIN coverage would be more cost-effective than IRS (economically dominant versus ICERs $114–2,008/DALY averted). In the central regions, changing from LLINs to IRS would not be cost-effective; in fact, it would be more costly and less effective (i.e., dominated). In the southern regions, at 80% coverage, LLINs were more costly and less effective than IRS, due to LLINs' low usage rate of 35%; thus, IRS effectively covers more of the population than LLINs (even at the same coverage level).

In general, LLIN campaign intervention costs were less costly than IRS campaigns (∼$2,200 for initial distribution at 80% coverage compared with ∼$4,850 per 1,000 persons over 3 years for IRS 80% coverage, Figure 3A–C). In the northern and central regions, LLIN campaigns resulted not only in lower intervention costs, but accrued lower direct and indirect costs than IRS campaigns. While the intervention costs were similar across regions, health-care costs and productivity losses differed due to differences in transmission and subsequent number of malaria episodes. In the southern regions, IRS campaigns accrued lower direct health-care costs (ranging from $9,584 to $13,672 per 1,000 persons) and productivity losses (ranging from $5,347 to $8,240 per 1,000 persons) compared with LLIN campaigns (direct costs ranging from $11,607 to $16,449 and productivity losses ranging from $7,140 to $10,199 per 1,000 persons) as they were associated with a low usage rate (35%).

Cost-effectiveness results were robust to changes in intervention costs. When LLINs cost 20% more and IRS cost 20% less ($3.30 and $1.66 per person protected, respectively), in northern regions LLIN campaigns remained dominant even though IRS campaigns became more cost-effective (ICERs ≤ $844/DALY averted) and switching from 80% LLINs to 80% IRS would now be cost-effective. The same trends held when IRS cost 20% more and LLINs cost 20% less, switching from LLINs to IRS was only cost-effective with 100% IRS coverage (ICER $927/DALY averted). In the central and southern regions of Mozambique, cost-effectiveness trends were unchanged, regardless of LLIN and IRS cost (±20%).

### Impact of LLIN usage.

Figure 4

**Figure 4.**
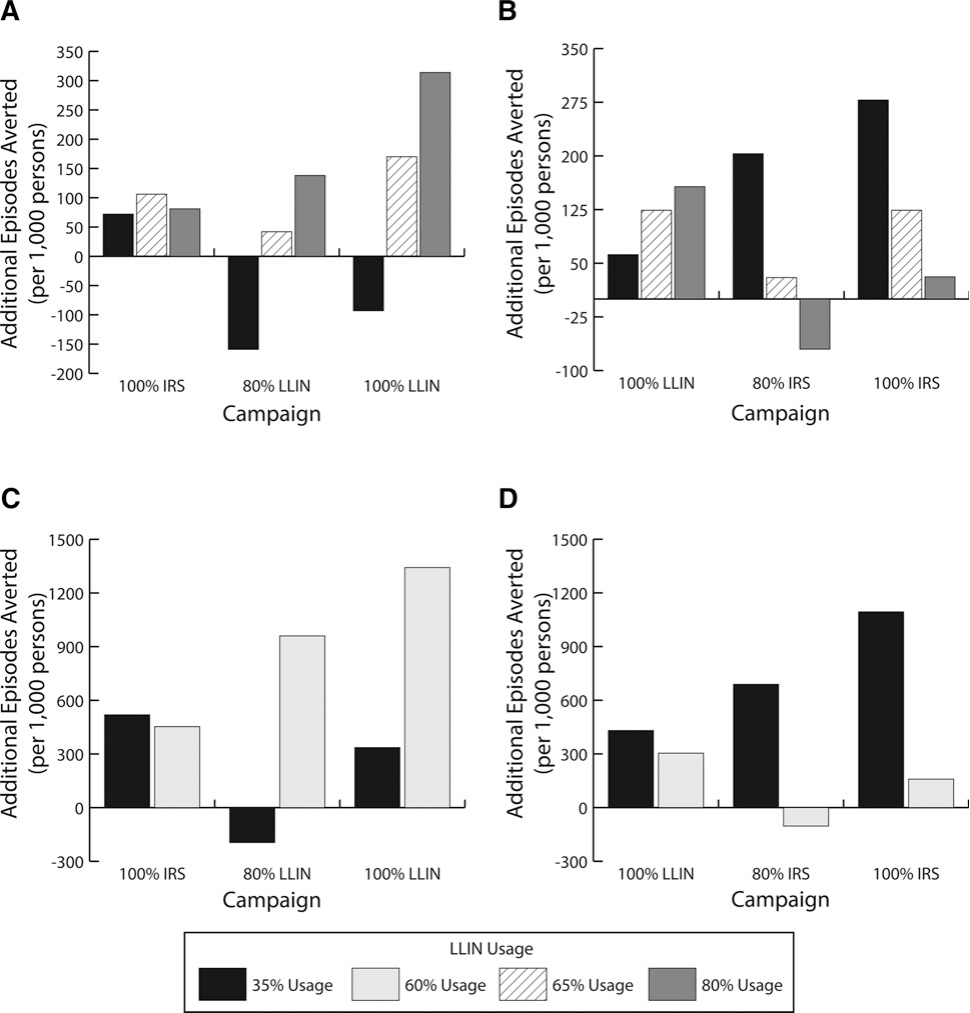
Impact of increasing long-lasting insecticidal net (LLIN) usage on the number of additional malaria episodes averted over 3 years for LLIN and indoor residual spraying (IRS) campaigns in the northern and southern regions of Mozambique. (**A**) Northern region with a baseline of 80% IRS coverage; (**B**) northern region with a baseline of 80% LLIN coverage; (**C**) southern region with a baseline of 80% IRS coverage; and (**D**) southern region with a baseline of 80% LLIN coverage.

and Table 4 show impact of LLIN usage in the northern and southern regions. When LLIN usage was lower, more gains (additional episodes averted) were accrued when switching to IRS (Figure 4B and D), whereas when LLIN usage was high, more gains were achieved by increasing LLIN coverage to 100% (Figure 4). Thus, with higher LLIN usage rates, the value of switching to an IRS campaign decreased and the cost-effectiveness of LLINs increased (Table 4). When LLIN usage was higher, the benefit of switching to IRS decreased (cost-effectiveness decreased compared with scenarios with lower usage rates). Higher LLIN usage also increased the cost-effectiveness of switching from IRS to LLINs. This is especially evident in the southern region, where an 80% LLIN campaign would be less costly and more effective (i.e., dominant) than 80% IRS with a 60% LLIN usage rate, but would be more costly and less effective (i.e., dominated) with a 35% LLIN usage rate (Table 4). Figure 4A and B and Table 4 also show what would happen with a substantial decrease in LLIN usage (from 65% to 35%) in areas of high transmission and high resistance (e.g., northern region). Under those conditions, switching from IRS to LLINs was never cost-effective.

### Impact of resistance.

As insecticide resistance is on the rise, we evaluated the impact of increasing the current vector resistance to pyrethroids in the central region (which is currently at a medium level), while holding all else constant. While maintaining an LLIN coverage of 80% (60% usage), increasing resistance from medium to high led to 99 additional episodes per 1,000 persons (3 DALYs/1,000 persons and 5 deaths/100,000 persons), generating an additional $706 in direct health-care costs and $771 in productivity losses. Despite this increase in the malaria burden, switching from LLINs to IRS (nonpyrethroid) was still not cost-effective. With a baseline of 80% IRS, switching to LLINs at either coverage level remained economically dominant.

In a high-transmission setting, increasing resistance reduced the effectiveness of LLIN campaigns (per 1,000 persons there would be approximately four more episodes, accruing five additional DALYs, and $259 and $1,289 in additional health-care costs and productivity losses, respectively). Switching to 80% IRS from 80% LLINs was still not cost-effective.

## Discussion

In Mozambique, increasing coverage of either IRS or LLINs was always cost-effective and in some cases increasing coverage of LLINs would be cost saving (i.e., would save costs in addition to averting DALYs). Our results show that one can usually justify increasing coverage by at least 20% (as in our modeled scenarios), but may vary for smaller increases (e.g., 90–95%). Our results vary by region, reflecting differences in malaria transmission, seasonality, current/historic interventions, and current resistance patterns and levels. In general, between the two interventions, LLINs were usually more efficacious and cost-effective except where LLIN usage was low, such as the southern region of Mozambique (35% usage). Increasing resistance to the insecticide used in LLINs decreased their effectiveness and thus increased the cost-effectiveness of IRS; however, LLINs were still better under modeled conditions. Thus, our results show that more tailored approaches are necessary to account for the heterogeneity of malaria epidemiology, even within a country.

Although LLINs tended to be more cost-effective, this does not necessarily suggest that LLINs should replace IRS in all cases. There is a great deal of social and institutional inertia that should be considered before changing the common practices. In particular, there are two factors in the presence of which IRS may still be more prudent. The first is low LLIN usage. Low LLIN usage may hinder even successful LLIN distribution campaigns if the population is not fully on board or are reluctant to change/use.^14^–^16^ A situation such as this may call for a behavior change campaign. IRS deployment in this context could serve to protect the population until such a time when they make more use of owned LLINs, if community acceptance of IRS is satisfactory. The second is high resistance against pyrethroids, especially since LLINs exclusively use pyrethroid insecticides. The use of nonpyrethroid insecticides and their spray rotation may help to reduce or delay vector resistance to these insecticides.^17^–^20^ Hence, other considerations also need to be take into account when tailoring approaches and planning malaria operation campaigns. Additionally, IRS acceptability should be considered as successful spraying programs rely on high compliance. Studies show people have mixed feelings about IRS and acceptability has been noted to be related to perceived benefits in the reduction of mosquitos, nuisance-biting, and malaria.^21^–^24^ However, a study in Mozambique found IRS to be broadly acceptable even at low levels of perceived efficacy based on a sense of citizenship.^25^

Our study highlights the importance of surveillance of LLIN usage and appropriate use. However, with constrained resources, it would be imperative to get a handle on LLIN usage rate as this is a major variable that impacts the value and effectiveness of LLIN campaigns. Not knowing the usage rate of LLINs in a region can substantially hamper the success of an LLIN distribution campaign. Having a sense of the usage rate given LLIN access can help program planners and decision-makers make appropriate decisions about intervention campaigns and implementation. Where appropriate usage is low, education programs should accompany LLIN distributions to ensure proper usage and promote good practices.^15^,^26^ Improper use of nets and human behavior may reduce their efficacy and duration of protection.^27^–^30^ In addition, our study shows the importance of monitoring insecticide resistance. The value of LLINs decreases with increasing resistance intensity. Without proper monitoring, LLIN campaigns may be inappropriate for malaria prevention and may put persons at higher risk. Additionally, further research and development is needed on other insecticides for use on nets. This could increase LLIN effectiveness while decrease the possibility for progressive development of resistance.

Our study illustrates the usefulness of models in determining the effects of decisions. Modeling and simulation can serve as virtual “policy laboratories” for public health officials, policy makers, and funders, and enables us to practically evaluate questions at hand. This in turn could save them considerable time, effort, and expense that trial and effort would bring. Modeling malaria intervention campaigns can determine potential impacts, benefits, and modifications. Additionally, removing or changing an intervention in the real world may be unethical by putting people at risk; modeling and simulation can overcome this problem and help fully explore the impact of changing interventions.

### Limitations.

By definition, all models are simplifications of real life,^31^ and as such cannot represent every event or outcome. Our model assumes conservative assumptions about the effectiveness of LLINs and optimistic assumptions about IRS (i.e., IRS was modeled with Actellic CS, one of the products with lowest documented resistance against its active ingredient). We assumed that all patients with severe disease were hospitalized and that patients with uncomplicated episodes were not. Additionally, we did not account for mortality from severe disease outside of the hospital. We also did not include any pre-referral treatment costs for those who may seek care at a lower level prior to hospitalization. We did not directly account for compliance with drug treatment due to variations depending on treatment drug and course and lack of reliable data. Model input data came from various sources of differing quality and was supplemented by expert opinion; thus, our results may be refined as more data become available. As our models were calibrated and populated with data specific to Mozambique, our results may not be generalizable outside of this country, unless other regions have similar malaria burdens and transmission patterns.

## Conclusions

Our simulation results show that LLINs were more cost-effective than IRS in the northern and central regions of Mozambique, and that it is worth considering prioritizing LLIN coverage and use. However, despite being less cost-effective, IRS may have an important role in insecticide resistance management and control of epidemics. Malaria intervention campaigns are not a one-size-fits-all solution, and tailored approaches are necessary to account for the heterogeneity of malaria epidemiology, even within a country.

## Supplementary Material

Supplemental Appendix.

## Figures and Tables

**Table 1:** Model input parameters, values, and sources

Parameter*	Costs (2014 $US)	Probabilities		
		0–59 months old	5–14 years old	≥ 15 years old
Gross national income per capita^13^	541			
IRS cost per person protected per year†§	2.08			
LLIN cost per person protected per net distribution (every 3 years)‡§	2.75			
RDT§	0.73	0.30	0.30	0.30
Microscopy§	0.90	0.0678	0.0678	0.0678
Care in rural area for uncomplicated malaria at				
Government facility^11^,^32^–^34^	4.65; 7.84¶	0.5871	0.5871	0.5871
Private facility^11^,^32^–^34^	6.51; 10.98¶	0.0013	0.0013	0.0013
CHW^11^	0.12	0.0158	0.0158	0.0158
Shop/healer^11^	0.51	0.0297	0.0297	0.0297
Other^11^	0.51	0.0271	0.0271	0.0271
Home/self-treatment^11^		0.19	0.19	0.19
No treatment^11^		0.15	0.15	0.15
Hospitalization for severe malaria^35^	72.73			
CM^36^,^37^		0.022	0.022	0.1392
Mortality from CM^36^,^37^		0.182	0.182	0.2308
Neurological sequelae^38^		0.0278	0.0278	0
SMA^36^,^37^		0.173	0.173	0.1646
Mortality from SMA^36^,^37^		0.057	0.057	0.0769
		Cure rates		
Treatments				
Artemether–lumefantrine^39^–^41^	1.2	0.763	0.904	0.904
Artesunate–amodiaquine^39^,^40^,^42^	0.047	0.889	0.9722	0.9722
Quinine^39^	0.506	1∥	1∥	1∥
Non-ACT antimalarial^43^–^45^	0.42	0.795	0.885	0.885
Nonmalarial drug		Probabilities		
Blood transfusion^37^,^46^	17.39	0.291	0.291	0
Progress to severe disease vs. naturally clear if not cured†		0.1	0	0
	Durations			
	All ages	0–59 months old	5–14 years old	≥ 15 years old
Missed productivity days^47^		2.9	3.41	3.41
Days sick/symptoms^47^		6.4	5.8	5.8
Anemia with transfusion**	2			
Anemia without transfusion**	14			
Neurological sequelae††	Lifelong			
Disability weights^48^				
Malaria episode	0.191			
Neurological sequelae	0.471			
Anemia	0.012			

ACT = artemisinin-based combination therapy; CHW = community health worker; CM = cerebral malaria; IRS = indoor residual spraying; LLIN = long-lasting insecticidal net; RDT = rapid diagnostic test; SMA = severe malaria anemia.

* Numbers after parameter are references for the given values.

† Total IRS budget with bendiocarb implementation costs doubled for proportion of population covered by bendiocarbs (13.9%) for the year in which they are planned to be sprayed (as sprayed twice year). This total was divided by the population to be covered by IRS (assuming a 2.5% population growth rate per year to determine this value for 2015 and 2016) to determine the cost per person protected by IRS. Additional costs per person sprayed were added to the resulting value to determine the total cost per person protected.

‡ Total cost per net divided by 1.82 persons per net.

§ Calculated from in country data sources.

¶ Cost for those under 5 years of age; cost for those ≥ 5 years of age.

∥ Assumed to be 100% (mortality rate applied before cure rate; those not dead assumed to be cured).

** Expert opinion.

†† Assumed to be lifelong, that any neurological outcomes caused irreversible damage.

**Table 2:** Number of additional malaria episodes, deaths, and DALYs averted by increasing coverage or changing intervention campaigns by region in Mozambique

	Northern region			Central region			Southern region		
	Additional episodes averted*	Additional deaths averted†	Additional DALYs averted*	Additional episodes averted*	Additional deaths averted†	Additional DALYs averted*	Additional episodes averted*	Additional deaths averted†	Additional DALYs averted*
Comparator: continue baseline of 80% IRS									
Increase to 100% IRS	106	7	3.9	292	10	6.5	519	15	8.9
Change to 80% LLIN	42	1	0.9	737	26	16.6	−195	−6	−3.8
Change to 100% LLIN	170	9	5.5	1,330	45	28.7	336	7	5.0
Comparator: continue baseline of 80% LLINs									
Increase to 100% LLIN	124	7	4.0	536	18	11.8	431	15	8.5
Change to 80% IRS	30	2	1.2	−462	−18	−10.9	689	24	13.5
Change to 100% IRS	123	9	5.0	−124	−4	−2.3	1,094	35	20.4

DALY = disability-adjusted life-year; IRS = indoor residual spraying; LLIN = long-lasting insecticidal net. Negative values imply additional cases, deaths, or DALYs; Scenarios maintain current insecticide resistance and LLIN usage rates in each region.

* Per 1,000 persons.

† Per 100,000 persons.

**Table 3:** Economic outcomes (ICER and cost per additional episode averted) over 3-year campaigns from the government perspective (all direct costs) by region in Mozambique

	Northern region	Central region	Southern region
ICER			
Comparator: continue baseline of 80% IRS			
Increase to 100% IRS	114*	Dominant	Dominant
Change to 80% LLINs	Dominant	Dominant	Dominated
Change to 100% LLINs	Dominant	Dominant	Dominant
Comparator: continue baseline of 80% LLINs			
Increase to 100% LLINs	Dominant	Dominant	Dominant
Change to 80% IRS	2,008†	Dominated	Dominant
Change to 100% IRS	594*	Dominated	Dominant
Cost per additional episode averted			
Comparator: continue baseline of 80% IRS			
Increase to 100% IRS	4.2	−2.3	−3.9
Change to 80% LLINs	−69.3	−10.1	NA
Change to 100% LLINs	−19.4	−8.0	−12.4
Comparator: continue baseline of 80% LLINs			
Increase to 100% LLINs	−2.5	−5.5	−5.1
Change to 80% IRS	82.4	NA	−2.5
Change to 100% IRS	23.7	NA	−2.7

ICER = incremental cost-effectiveness ratio; IRS = indoor residual spraying; LLIN = long-lasting insecticidal net; NA = intervention did not avert any additional cases compared with baseline. Scenarios maintain current insecticide resistance and LLIN usage rates in each region.

* Highly cost-effective.

† Not cost-effective.

**Table 4:** Impact of LLIN usage on the ICER in the northern and southern regions of Mozambique from the government perspective (all direct costs)

	Campaign			
	80% IRS	100% IRS	80% LLINs	100% LLINs
Northern region				
Comparator: continue baseline of 80% IRS				
35% usage	–	205†	Dominated	Dominated
65% usage*	–	114†	Dominant	Dominant
80% usage	–	348†	Dominant	Dominant
Comparator: continue baseline of 80% LLIN				
35% usage	158†	158†	–	30†
65% usage*	2,008‡	594†	–	Dominant
80% usage	Dominated	1,319	–	Dominant
Southern region				
Comparator: continue baseline of 80% IRS				
35% usage*	–	Dominant	Dominated	Dominant
60% usage	–	Dominant	Dominant	Dominant
Comparator: continue baseline of 80% LLIN				
35% usage*	Dominant	Dominant	–	Dominant
60% usage	Dominated	1,212	–	Dominant

ICER = incremental cost-effectiveness ratio; IRS = indoor residual spraying; LLIN = long-lasting insecticidal net.

* Current LLIN usage rate in region.

† Highly cost-effective.

‡ Not cost-effective.
