# Supplementary material for: The Economic Value of Long-Lasting Insecticidal Nets and Indoor Residual Spraying Implementation in Mozambique
Source: Am J Trop Med Hyg. 2017 Jun 7;96(6):1430–40. doi: 10.4269/ajtmh.16-0744 (PMC5462583; doi:10.4269/ajtmh.16-0744)
Supplement: Supplementary file 1 [file SD7.pdf]

SUPPLEMENTAL APPENDIX

JANUS MALARIA MOZAMBIQUE MODEL

Each new malaria case generated by the transmission model then feeds into JANUS's malaria clinical outcomes probability tree (Figure 1) that determines the clinical outcomes and associated costs of each case. Each case has a probability of being symptomatic or asymptomatic. Each symptomatic case has a probability of being uncomplicated or complicated. Uncomplicated malaria episodes could either seek care or not seek care. Those seeking care have probabilities of visiting a government health facility (i.e., clinics, hospitals, or dispensaries), private health facility, a community health worker (CHW), a shop or traditional healer, other source, or they can stay home or self-treat (Table 1). Individuals seeking care at a government or private facility have probabilities of being diagnosed with the use of microscopy or a rapid diagnostic test (RDT), depending on availability. Those seen by a CHW are diagnosed with RDTs if available. All those seeking care at one of these three types of facilities are treated for malaria, regardless of diagnostic test result, reflecting the tendency for malaria to be overtreated and the presumptive treatment of negative test results based on clinical suspicion. Treatment followed Mozambique's guidelines and recommended drug regimens. Those seeking care at a government or private health facility receive either artemether–lumefantrine (AL) or artesunate–amodiaquine (ASAQ) and we assumed 95% received AL and the remaining 5% received ASAQ (based on in-country data). Those seen by a community health worker (locally known as an agente polivalente elementare, APE). Those seeking care at a shop or traditional healer receive a non-artemisinin-based-combination-therapy antimalarial drug (30% of persons) or non-antimalarial (70% of persons) and those who stay home or self-treat do not take an antimalarial drug. Each drug treatment regimen has a probability of cure; when the treatment is not successful, persons have a probability of progressing to severe disease and are subsequently hospitalized; the remaining undergo spontaneous clearance of parasitemia.

All those with severe episodes are hospitalized and have probabilities of experiencing cerebral malaria (CM) and severe malaria anemia (SMA); all other episodes are categorized as severe. CM patients have a probability of mortality and of developing lifelong neurological sequelae if surviving. SMA patients have a probability of receiving a blood transfusion, which shortened the duration of anemia. If no transfusion is received, anemia eventually self-resolves. All SMA patients, regardless of if a transfusion is received, have a probability of mortality. All severe episodes are treated with quinine.

Each episode accrues direct and indirect costs of illness based on its health outcomes. Direct costs include the cost of health care (i.e., health facility visits and hospitalization) and treatment, whereas indirect costs include productivity losses for work absenteeism and mortality due to malaria infection. Productivity losses due to work absenteeism are based on lost productivity days due to illness, or in the case of a child, productivity time lost for an adult caring for the child, and the daily wage for Mozambique.[1] The cost of mortality is the net present value of lifetime productivity losses based on a person's remaining life expectancy[2] and annual wages.[1]

CURRENT CIRCUMSTANCES IN MOZAMBIQUE

The seasonality of malaria transmission was tuned to be representative for Mozambique in the OpenMalaria model and was fit using monthly incidence data from Mozambique. Three vector species in Mozambique were modeled according to their relative numbers as follows: *Anopheles gambiae* (36.65%), *Anopheles funestus* (41.61%), and *Anopheles arabiensis* (21.74%). Resistance levels to pyrethroids were calibrated based on data in the literature from Pitoa, Cameroon, and Zeneti, Tanzania, for PermaNet 2.0 (Vestergaard, Lausanna, Switzerland). We assumed resistance levels of 77% and 30% for high and low resistance, respectively.

We assumed Actellic (pirimphos methyl) CS for indoor residual spraying (IRS) and PermaNet 2.0 for long-lasting insecticidal nets (LLINs) and constant usage rates given ownership over time. If deployed, the timing of LLINs and IRS was at the beginning of the year. This represents an optimal timing of deployment, especially for IRS, as the effect of the insecticide is strongest just before the main transmission peak. In Mozambique, transmission starts to peak at 3 months. OpenMalaria assumes net decay over time with attrition half-life and hole formation. The parameterization of the effect of holes and insecticide in nets on protection against biting by and killing of mosquitoes, depending on the mosquitoes' resistance status, has been described in detail in previous work. The parameterization for IRS was based on a study that lasted 1 year, thus allowing to assess and model the shape and rate of decay over time.

SUPPLEMENTAL REFERENCES

1. The World Bank, 2014. *World Development Indicators, 1960–2013*. Washington, DC: The World Bank. Accessed October 2014.
2. World Health Organization, 2013. *Global Health Observatory: Country Statistics*. Geneva, Switzerland: World Health Organization. Available at: http://www.who.int/gho/countries/en/#M. Accessed May 12, 2015.
